# An algorithmic approach of reconstruction for cranioplasty failure: A case series

**DOI:** 10.1097/MD.0000000000033011

**Published:** 2023-02-22

**Authors:** Yu-Chi Wang, Yi-Chia Wu, Chao-Wei Chang, Chia-Li Chung, Su-Shin Lee

**Affiliations:** a Division of Plastic and Reconstructive Surgery, Department of Surgery, Kaohsiung Medical University Hospital, Kaohsiung, Taiwan; b Department of Surgery, Faculty of Medicine, College of Medicine, Kaohsiung Medical University, Kaohsiung, Taiwan; c Regenerative Medicine and Cell Therapy Research Center, Kaohsiung Medical University, Kaohsiung, Taiwan; d Division of Neurosurgery, Department of Surgery, Kaohsiung Municipal Siaogang Hospital, Kaohsiung, Taiwan.

**Keywords:** case series, complication, cranioplasty, failure, reconstruction

## Abstract

**Rationale:**

Cranioplasty is a surgical procedure used to repair cranial defects for both cosmetic and functional reasons. The complication rate of cranioplasty is between 10% and 50%. The failure of cranioplasty is associated with various factors, including etiologies, types of material, and the timing of cranioplasty. In this study, a case series of managing cranioplasty complications at a single institution.

**Patient concerns:**

Eighteen patients were identified who underwent craniofacial defect reconstruction due to the failure of their initial cranioplasty between January 2010 and May 2020. Five men (27.78%) and thirteen women (72.22%) were included. The mean age was 39.61 years old. The average follow-up duration was 5.94 years.

**Diagnoses:**

The indication for initial cranioplasty included previous decompressive craniectomy (77.78%, n = 14), traumatic cranial defects (16.67%, n = 3), and congenital cranial deformity (5.56%, n = 1). The reported complications were infection (50%, n = 9), implant exposure (50%, n = 9), wound dehiscence (22.22%, n = 4) and cranial deformity (11.11%, n = 2).

**Interventions:**

More than half of the materials used for initial cranioplasty were synthetic [titanium mesh: 44.44%, n = 8; polymethyl metacrylate: 5.56%, n = 1; titanium mesh and polymethyl metacrylate: 5.56%, n = 1], while 44.44% of the patients received autologous bone graft.

**Outcomes:**

Of all reconstructive procedures for cranioplasty failure, 55.56% was local flap with or without skin graft (n = 10), 16.67% was free flap (n = 3), 11.11% was skin graft only (n = 2), 5.56% was regional flap (n = 1). The free flap survival rate was 100% (3/3), and implant removal with sebsquent second cranioplasty was performed on 27.78% (n = 5) of the patients.

**Lessons:**

Management of cranioplasty failure can be challenging due to infection, refractory implant exposure, and wound dehiscence. The principles of management are based on adequate infection control and reconstructive ladder. Meanwhile, collaboration with plastic surgery and neurosurgery should be strengthened in order to achieve the best clinical outcomes.

## 1. Introduction

Cranioplasty is a crucial procedure to reconstruct cranial defects caused by skull fractures, life-saving decompressive craniectomy, and congenital cranial anomalies.^[1,2]^ Among these indications, cranioplasty is particularly necessary after decompressive craniectomy. After removing the large skull flap, the force of the atmosphere pressure on the unprotected cranium resulted in dysregulation of cerebrospinal fluid and cerebral blood flow.^[3,4]^ This subsequently causes neurologic deficits known as trephined syndrome, also referred as sinking skin flap syndrome.^[4]^ The longer the wait for cranioplasty, the greater the risk of developing trephined syndrome.^[1]^ Furthermore, the patients with skull deformities are subjected to psychological stress and have a low quality of life.^[5]^ As a result, cranioplasty not only protects cerebral structures but also preserves the cranial contour for cosmesis and psychosocial function. However, according to the literature, the complication rate of cranioplasty ranges from 10% to 50%.^[1,6,7]^ Early failure includes surgical site infection and hematoma, which typically occur 3 to 7 days postoperatively. While the delayed failure commonly manifests months or years after cranioplasty, which involves wound dehiscence, bone resorption, and implant exposure.^[6]^

Because of the intricate anatomy of the scalp and cranium, it is unique in treating cranioplasty failure. From outer to inner, the scalp is composed of 5 soft tissue layers: skin, subcutaneous tissue, galea aponeurotica, loose areolar tissue, and pericranium.^[8]^ The cranium bone is located beneath the scalp.^[8,9]^ Vessels, nerves, and lymphatic drainage run in the subcutaneous tissue and just superficial to the galea aponeurotica, which is critical for considering the vascularity and innervation for scalp local flap planning.^[10]^ The galea aponeurotica is an inelastic fibrotic tissue that provids strength, while the underlying loose areolar tissue is responsible for scalp mobility.^[8]^ The interweaving of tight galea aponeurotica and loose areolar tissue contributes to the formation of tight and loose regions of the scalp.^[11]^ The scalp flap is usually raised in the layer of loose areolar tissue because it can be dissected with less effort and without injuring vital neurovascular structures.^[10]^ Pericranium is the innermost layer of the scalp.^[8,9]^ The pericranium keeps the blood supply of the cranium bone beneath and serves as a vascularized surface for a skin graft.^[8]^ Thus, In patients who underwent cranioplasty, the formation of fibrotic scar tissue and the proliferation of granulation tissue in the scalp wound make the cranioplasty revision more difficult than simple scalp reconstruction.

The cranium, commonly known as the skull or calvarium, is composed of frontal, parietal, temporal, occipital, and sphenoid bones.^[9]^ The cranium bone is comprised of 3 layers: an outer table, a central diploic space, and an inner table.^[10]^ The outer table is a thick cortical bone that is tightly attached to the pericranium, while the inner table is brittle and thin.^[9]^ In between the outer and inner tables, the central diploic space is filled with cancellous bone and small valveless vessels.^[8–10]^

According to studies, the cranioplasty revision rate can reach 26%,^[1,12]^ necessitating multiple interventions such as debridement, cranioplasty implant removal, cranioplasty revision, and scalp reconstruction.^[6,13,14]^ These findings suggest that managing cranioplasty failure is challenging. However, there is no consensus on the best strategy for managing cranioplasty failure. Various factors, including comorbidity, cause of failure, infection, defect size, and hairline involvement, should be carefully considered before the reconstructive planning. Therefore, the goal of this retrospective cohort study is to develop an algorithmic approach for cranioplasty failure management based on our 10-year experience with cranioplasty complications and the following managements.

## 2. Patients and methods

### 2.1. Study design

This retrospective study was conducted at a single medical center from January 2010 to May 2020 and was approved by the Institutional Review Board and Ethics Committee of Kaohsiung Medical University Hospital [KMUHIRB-E(I)-20210034]. The inclusion criteria were: patients who underwent cranioplasty due to craniectomy, craniofacial fracture, or congenital craniofacial anomaly; patients who had failures of cranioplasty, including infection, material exposure, wound dehiscence, poor healing wounds, and flap failure. Patients who had previously received radiotherapy in head and neck region were excluded. A total of 210 cranioplasty cases were screened, 18 cases were included. The patient following variables were collected, including: age; sex; indication of previous craniectomy; time between previous craniectomy and cranioplasty; number of reconstructive surgeries before definitive cranioplasty; material of definitive cranioplasty; cause of cranioplasty failure; follow-up duration; and scalp and skull defect size.

### 2.2. Assessment and management

The patients with cranioplasty failure were assessed with physical examinations, wound or abscess cultures, and computed tomography scans. Physical examinations were performed to thoroughly examine the conditions associated with cranioplasty failure, such as focal signs of infection, pus or abscess formation, wound dehiscence, flap necrosis, implant exposure, and skull deformity due to implant displacement or bone flap resorption. The wound or abscess cultures were collected when infection was suspected. A 3-dimensional virtual model of a computed tomography scan (General Electric Medical Systems, Milwaukee, WI) was used to measure the defect size.

### 2.3. Statistical analysis

The data were collected, statistical analysis was performed using SPSS (SPSS Inc., Chicago, IL). Percentages were calculated, continuous variables were expressed as the mean and range, or standard deviation. One-way analysis of variance was used to determine the significance between groups. A *P* value of < .05 was regarded as significant.

## 3. Results

### 3.1. Demographic features

Between January 2010 and May 2020, 210 patients who undergone decompressive craniectomy were identified. The study included eighteen of these cases who met the criteria.

The patient demographics are demonstrated in Table 1. The patients were 5 men and 13 women, with an average age of 39.61 years (ranging from 16–68 years) and a 5.94-year average follow-up (ranging from 2–10 years). The indication for initial cranioplasty included previous decompressive craniectomy (77.78%, n = 14), traumatic cranial defects (16.67%, n = 3), and congenital cranial deformity (5.56%, n = 1). The average timing of the first cranioplasty was 5.93 months after the previous craniectomy. More than half of the initial cranioplasty materials were synthetic [titanium mesh: 44.44%, n = 8; polymethyl metacrylate (PMMA): 5.56%, n = 1; combined titanium mesh and PMMA: 5.56%, n = 1], while autologous bone graft was used by 44.44% of the patients (n = 8). The overall failure rate for cranioplasty was 8.57% (n = 18 out of 210). The reported complications were infection (50%, n = 9), implant exposure (50%, n = 9), wound dehiscence (22.22%, n = 4), and cranial deformity (11.11%, n = 2).

**Table 1:** Patient profile and outcomes

Case No.	Age	Sex	Indication of previous craniectomy/operation	Timing of first cranioplasty (mo)	Cause of cranioplasty failure	Timing of complication occurred post operation (w)	Material of Primary cranioplasty	Soft tissue reconstruction	Material of secondary cranioplasty	Implant removal	Follow-up (yr)	Scalp/skull defect size (cm^2^)
1	19	M	Congenital plagiocephaly	N/A	Skull deformity	61	Titanium mesh	Fat graft	Autologous bone graft from rib	No	10	Nil/ 23.99
2	43	M	Traumatic SDH	9	Infection implant exposure	4	Titanium mesh	Free flap	Nil	Yes	10	30.8/ 106.59
3	26	F	Traumatic EDH	6	Infection implant exposure	11	Titanium mesh	Free flap	Nil	Yes	6	49.6/ 66.52
4	27	F	Traumatic SAH	6	Infection implant exposure	11	Titanium mesh	Skin graft	Nil	No	3	2.7/ 99.90
5	40	F	Traumatic SDH	6	Implant exposure	19	Titanium mesh	Local flap + skin graft	Nil	No	3	15/ 50.77
6	60	F	Spontaneous SAH	4	Implant exposure	20	Titanium mesh	Local flap + skin graft	Nil	No	2	14.74/ 96.71
7	61	F	meningioma	2	Infection implant exposure	20	Titanium mesh	Local flap + skin graft	Nil	Yes	3	8.4/ 88.91
8	16	F	Traumatic SDH	9	Implant exposure	8	Customized 3D titanium mesh	Free flap	Nil	No	2	32.2/ 100.53
9	45	F	Meningioma	6	Infection implant exposure	12	Titanium mesh and PMMA	Local flap	Titanium mesh	No	8	6.25/ 53.47
10	67	F	Spontaneous SAH	6	Infection	2	PMMA	Skin graft	Autologous bone graft	Yes	9	22.77/ 61.65
11	36	M	Frontal bone fracture	N/A	Skull deformity	N/A	Autologous skull graft	Fat graft	Titanium mesh	No	10	Nil/ 32.68
12	40	M	Spontaneous SAH	4	Infection	13	Autologous skull graft	Local flap	Nil	No	8	39/ 37.44
13	19	F	Traumatic SAH	2	Wound dehiscence	8	Autologous skull graft	Regional flap	Nil	No	2	10/ 31.40
14	23	F	Frontal bone fracture	N/A	Skull deformity	72	Autologous skull graft	Local flap	Nil	No	6	4.62/ 86.55
15	26	F	Traumatic SDH	7	Wound dehiscence Infection implant exposure	60	Autologous skull graft	Local flap	Nil	No	2	8/ 93.89
16	36	M	Traumatic SDH	7	Infection	4	Autologous skull graft	Local flap	Titanium mesh	Yes	8	8/ 116.54
17	61	F	meningioma	9	Flap breakdown	29	Autologous skull graft	Local flap + skin graft	Nil	No	5	3.6/ 73.16
18	68	F	Frontal bone fracture	N/A	Flap breakdown	60	Autologous skull graft	Local flap + skin graft	Nil	No	10	13.8/ 39.25

PMMA = polymethyl metacrylate.

### 3.2. Outcomes

**Figure 1.**
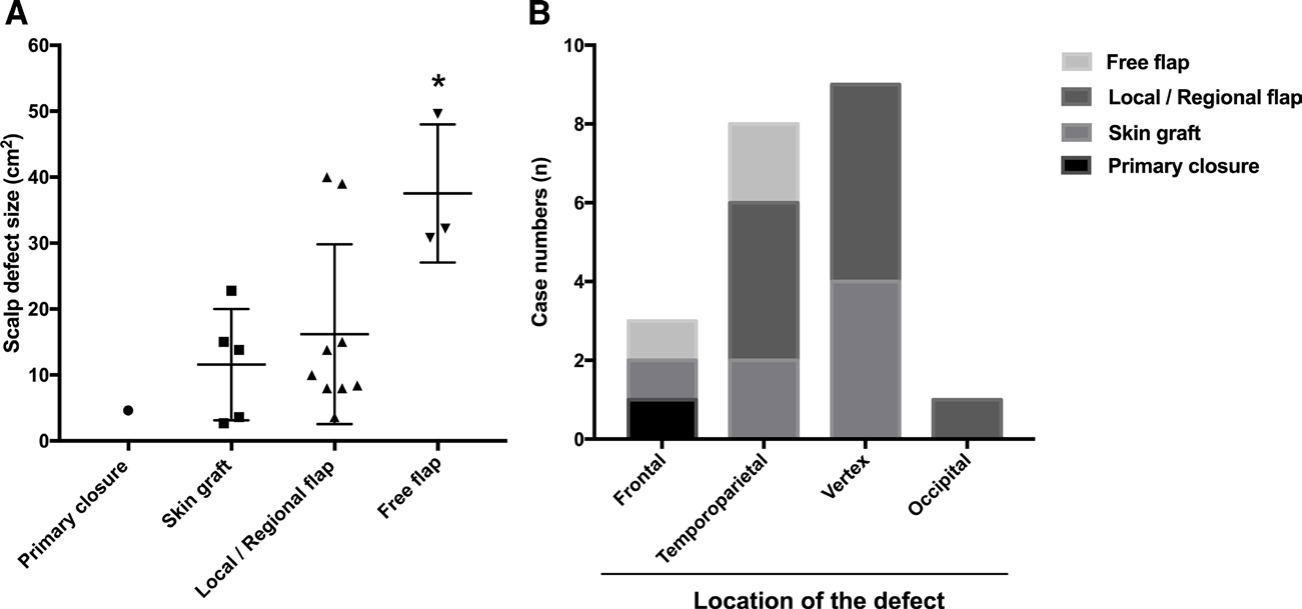
Analysis of defect classification and reconstructive procedures. (A) The association between scalp defect size and the reconstructive procedures. For scalp defect size larger than 30cm^2^, the patients underwent free flap surgery. *, *P* < .05. (B) The association between location of scalp defect and the reconstructive procedures. The majority the scalp defects were in the vertex region, and these patients had a local/regional flap or skin graft performed. The second most affected location was the temporoparietal region, and about half of these defects were treated with local/regional flap.

The average numbers of reconstructive surgeries for previously failed cranioplasty was 4.11. Of all these reconstructive surgeries for cranioplasty failure, 55.56% was local flap with or without the combination of skin graft (n = 10), 16.67% was free flap (n = 3), 11.11% was skin graft alone (n = 2), 5.56% was regional flap (n = 1) (Table 1). The survival rate of free flap was 100% (3/3). The analysis of scalp size and reconstructive procedures showed that patients with the smaller scalp defect sizes had primary closure, skin graft or local/regional flap, whereas patients with the larger scalp defect sizes underwent local flap, regional flap, or free flap reconstruction (Fig. 1A). The most commonly affected location of cranioplasty failure was vertex, followed by temporoparietal, frontal and occipital regions (Fig. 1B). A skin graft and a local/regional flap could be used to repair the vertex defect. The temporoparietal defect was treated primarily with a local/regional flap. frontal and temporoparietal defects could be repaired with free flaps (Fig. 1B).

The overall implant removal rate was 27.78% (n = 5). 60% (n = 3) of these cases had artificial implant removed and 40% (n = 2) had autologous bone grafts removed. The reasons for implant removal were abscess formation (80%, n = 4) and recurrent wound infection (20%, n = 1). Secondary cranioplasty was performed on all patients who had a previous implant removed. Two patients received autologous bone grafts, and 3 patients had titanium mesh as the new implant (Table 1).

### 3.3. Case summary

#### 3.3.1. Case no. 3 of Table 1.

**Figure 2.**
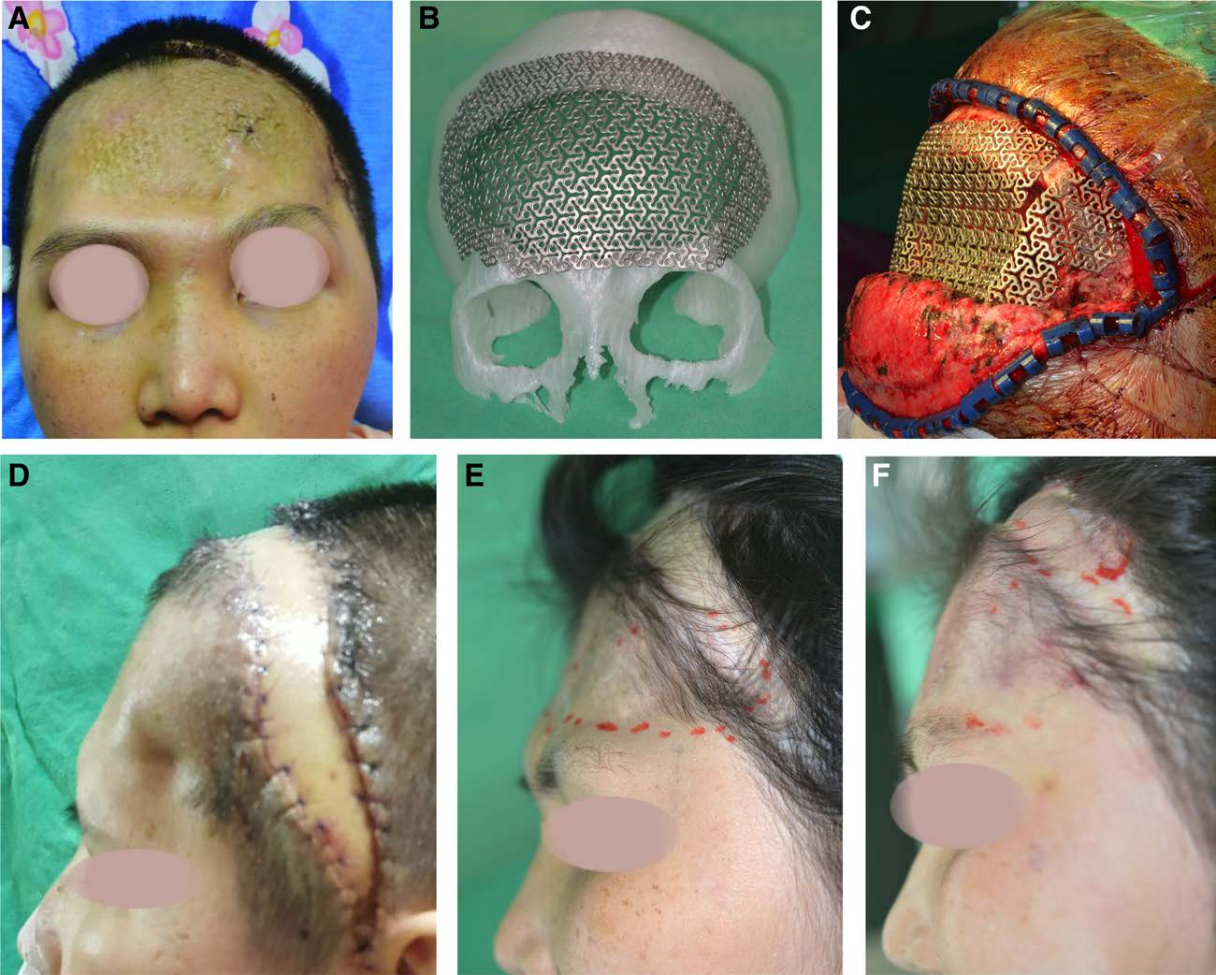
A 26-year-old woman with frontal implant exposure after cranioplasty (Case No. 3 of Table 1). (A) The preoperative image showed frontal implant exposure. (B) Customized 3-dimensional titanium mesh. (C) Intraoperative image. The first-stage reconstruction of titanium mesh reshaping and ALT flap for the scalp defect were performed. (D) Postoperative image took 1 week after the surgery. (E) The postoperative image was taken 5 months the surgery. A depression in the frontal region was noted. (F) Immediate postoperative image. The second-stage reconstruction of autologous fat graft for frontal depression was performed 5 months after the first-stage of reconstruction.

A 26-year-old woman suffered a frontal bone fracture and subdural hemorrhage due to a traffic accident. Cranioplasty with titanium mesh was performed 5 months after the decompressive craniectomy, frontal implant exposure was observed 8 months later (Fig. 2A). The size of skull defect was 66.52 cm^2^, the size of the skin defect was 16 cm^2^ in the frontal area. For preoperative planning, a customized 3-dimensional titanium mesh was created (Fig. 2B). The reconstruction was done in 2 stages. The first-stage of reconstruction was done with a new, reshaped titanium mesh and anterolateral thigh (ALT) flap for the scalp defect (49.6 cm^2^ in size) within the hairline (Fig. 2C). The patient recovered uneventfully with no implant exposure or other major complications. The patient is shown 1 week (Fig. 2D) and 5 months (Fig. 2E) after the first-stage of reconstruction. However, depression and irregular contour in the frontal region were still observed. Five months after the first-stage of reconstruction, the second-stage reconstruction was done with fat transplantation (50 mL of autologous fat graft from the abdomen) for the frontal contour defect (Fig. 2F). The patient reported a satisfactory outcome of the contour defect.

#### 3.3.2. Case no. 9 of Table 1.

**Figure 3.**
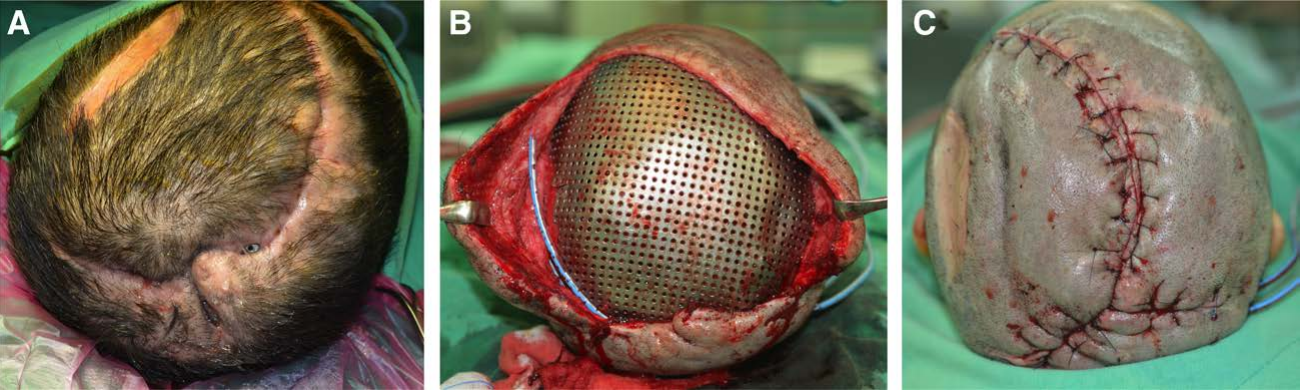
A 45-year-old woman with cranioplasty wound dehiscence and implant exposure (Case No. 3 of Table 1). (A) Preoperative image. (B) Intraoperative image. Titanium mesh reshaping and the scalp reconstruction with rotation flap. (C) Postoperative image was taken immediately after the surgery.

A 45-year-old woman with a history of meningioma had the tumor removed. Two months after cranioplasty with titanium mesh, she experienced wound dehiscence and implant exposure (Fig. 3A). The skull defect size measured 53.47 cm^2^ and the 2 scalp defect sizes were 2.25 cm^2^ and 4 cm^2^ in the vertex, respectively. The titanium mesh was revised, as well as the scalp rotation flap (Fig. 3B and C). During the postoperative follow-up, no implant exposure, wound dehiscence, or other major complication were observed.

## 4. Discussion

Our study reviewed 18 patients with cranioplasty failure and the outcome of the management in a 10-year, single-center, and consecutive cohort. We found that the cranioplasty failure rate was 8.57%, requiring varying degrees of reconstructive management. Similarly, the revision rate of cranioplasty was reported to be between 5% and 26% in the literature.^[1,13,15,16]^ Our cohort showed that, the leading causes of cranioplasty failure were infection (50%, n = 9) and implant exposure (50%, n = 9), followed by wound dehiscence (22.22%, n = 4) and skull deformity (11.11%, n = 2) (Table 1). These results were consistent with the findings of Sahoo et al^[6]^, who found that the most common cause of cranioplasty failure was infection (43%, n = 6 out of 14) and flap breakdown (21%, n = 3 out of 14). According to another retrospective cohort study, infection accounted for 72% (n = 8 out of 11) of the reasons for failed cranioplasty, while implant resorption (27%, n = 3 out of 11) was the second most common reason.^[13]^

The factors contributing to infection after cranioplasty are controversial among different study cohorts. Vijfeijken et al^[17]^ examined 276 consecutive patients who underwent autologous cranioplasty following decompressive craniectomy, and discovered that neoplasm as an indication for initial decompressive craniectomy was significantly correlated with infection after cranioplasty. The patients with brain tumors or neoplasms had poor surgical wound healing and an increased risk of infection, particularly those with tumor recurrence and radiotherapy. Another retrospective study of 103 patients with cranioplasty from subcutaneously preserved bone flaps found that infection is significantly associated to history of traumatic brain injury, as well as large defect size with maximum diagonal length > 11.07 cm.^[18]^ The patients underwent decompressive craniectomy due to traumatic intracranial hemorrhage in conjunction with scalp laceration and skull fracture usually resulted in wound infection.^[17,18]^ Furthermore, the greater the defect, the higher the risk of wound dehiscence. However, no significant difference in infection rate was found between the presence of brain tumor or neoplasm.^[18]^ Other risk factors for postcranioplasty infection have been identified as postoperative subdural fluid accumulation, wound dehiscence, the use of an autologous bone flap, the number of previous operations being < 3, and immunocompromised status.^[19,20]^ In our study, 5 of 12 traumatic brain injury cases and 2 of 3 meningioma cases had infection after cranioplasty, which was comparable other studies.^[17–20]^

**Figure 4.**
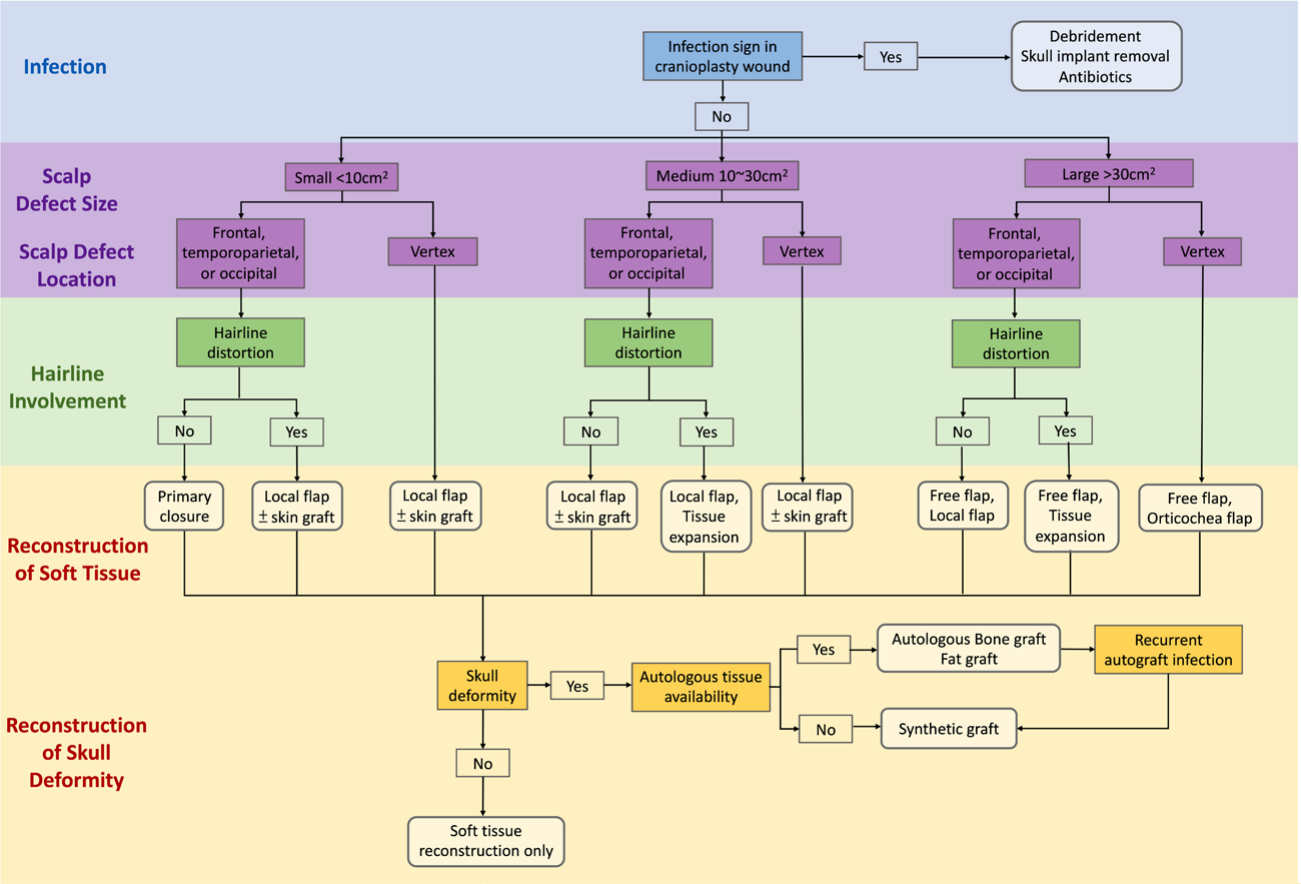
The algorithm for management of cranioplasty failure. The strategy for reconstruction of cranioplasty failure should follow the principles of the reconstructive ladder and the concept of “like with like” in order to achieve satisfying functional and aesthetic outcomes. The location and size of the defect, etiology, wound quality, infection, underlying tissue exposure, hairline involvement, and presence of skull deformity should all be carefully considered. Primary closure can be used for small defects in the loose region of the scalp. Local flap with or without skin grafting has been used for small defect with hairline involvement, medium-to large-sized scalp defects. Free flaps are indicated in medium-to large scalp defects with implant or cranial exposure and poor healing wounds. The choice of synthetic or autologous materials to treat skull deformity is determined by the infection condition and material availability.

The management of cranioplasty failure is usually exhausting, with multiple revisions required. According to the literature, the majority of cranioplasty patients undergo 3 to 4 operations for revision or reconstruction.^[21]^ In our cohort, the average numbers of reconstructive surgeries for previously failed cranioplasty was 4.11. Local flaps (55.56%, n = 10) are the most commonly used reconstructive procedure, usually in combination with a skin graft at the donor site of the local flap. Other procedures included free flap (16.67%, n = 3), skin graft alone (11.1%, n = 2), and regional flap (5.56%, n = 1) (Table 1). Although there is no agreement on how to manage cranioplasty failure, adhering to the principles of the reconstruction ladder can result in a successful reconstructive outcome. The reconstruction for cranioplasty failure is based on the reconstruction ladder and the concept of “like with like” to achieve a functional goal and cosmetic consideration. The algorithms are created based on the location and size of the defect, etiology, wound quality, underlying tissue exposure, hairline involvement, and presence of skull deformity (Fig. 4).

In general, primary closure can be used for small defects in the loose region of the scalp. A proper wound closure is achieved by a 2-layered closure and the approximation of the galea, which is a feasible layer of the scalp, to provide sufficient strength.^[11]^ Reduced tension decreases the risk of iatrogenic alopecia.^[10]^ To reduce the scalp wound edge bleeding and provide hydrodissection to avoid vascular injury, we prefer local anesthetic with an epinephrine-containing lidocaine solution in the incision wound for hemostasis. Hemostatic clips for scalp bleeding are avoided.

Skin grafting, including split- and full-thickness skin grafting, has been used for medium-to large-sized scalp defects, with the donor site in the rotational/advancement flap. The local flaps, including advancement, rotation, transposition flaps, and have been widely applied for scalp reconstruction owing to their reliability and low complication rate.^[22]^ The principles of local flap design include a wide flap base, preserving the scalp vascularity and hairline, and minimizing the number of flaps.^[10,11]^ Undermining should be considered to achieve tension-free wound.^[23]^ Advancement flaps are typically used to repair small defects in relatively loose areas such as the temporoparietal region. The rotational flap should be 4 to 6 times as long as the original scalp defect. For medium-to large-sized vertex defects, the O to Z flap and Ortichoa flap, are indicated.^[10,24,25]^ In contrast, regional flap has played a smaller roles in scalp reconstruction in recent decades, and it is only considered when the peripheral tissues are insufficient for local flap and the patient condition cannot tolerate the operation of free tissue transfer.

Microvascular free flaps are indicated in medium-to large scalp defects with implant or cranial exposure and poor healing wounds. The latissimus dorsi, radial forearm, gracilis, and parascapular flaps are among the free flaps available to the donor.^[10,26–28]^ We preferred the anterolateral thigh (ALT) flap as a donor for free tissue transfer due to suitable caliber vessels for anastomosis and small volume of the subcutaneous fat tissue.^[10,26]^ According to the location of the defect, candidate recipient vessels are superficial temporal, facial, and external jugular vessels.^[26,29]^ Despite the reliability of free flap reconstruction for scalp defects, iatrogenic alopecia and the bulky contour are the most concerning issues.^[10,26–29]^ A thorough preoperative discussion and consultation are required to establish rapport with the patient.

There 2 types of cranioplasty materials: biologic and synthetic. Autologous graft, allograft, and xenograft are the 3 types of biological materials.^[2,30]^ Because of their relatively low cost, autologous grafts are used widely used. The autologous graft can be derived from previously removed cranial bone, ribs, iliac crest, tibial bone, and so on.^[2,15,17,30]^ However, the most serious disadvantage of autologous grafts is the risk of bone resorption, which should be avoided in pediatric patients due to the high resorption rate.^[2,15,17]^ Furthermore, the graft infection rate is higher than that of synthetic materials. Because of concerns about infection, resorption, host immune responses, other biologic materials, including xenografts and allografts, and are less commonly used.^[2,31]^ In our study, 44.44% (n = 1) of the cases had cranioplasty with an autologous graft, and only 1 patient had a postcranioplasty infection. This patient underwent cranioplasty revision with titanium mesh, and no infection was observed during postoperative follow-up.

Synthetic grafts are made from a variety of materials. Polymethyl methacrylate (PMMA), hydroxyapatite, titanium mesh, and polyether ether ketone (PEEK) are the commonly used materials.^[7]^ Overall, synthetic materials have a lower rate of graft infection, resorption, and revision when compared to the biological bone grafts.^[2,32]^ Furthermore, with the advancement of computer-assisted 3-dimensional printing techniques, the use of custom-made synthetic grafts not only reduces operation time and failure rate but also provides satisfying cosmetic contours.^[2,33]^ However, some issues should be carefully considered before deciding on the optimal synthetic materials. PMMA accommodates skull growth poorly and should not be used in pediatric patients.^[34]^ Hydroxyapatite is a naturally occurring component of human bone with high biocompatibility. The low tensile property, on the other hand, increases the risk of fragmentation.^[12,35]^ Titanium mesh has a low infection rate, is biocompatibility, and can be combined with other synthetic materials.^[16,20]^ However, the nature of heat conduction and the artifacts on image studies are disadvantages.^[2]^ PEEK is light-weighted and nonheat conductive. It can be designed with high accuracy using the 3-dimensional printing technique, making it suitable for defects involving the fronto-orbito-temporal area.^[36,37]^ The only drawbacks are that PEEK has limited osteointegration and is costly.^[2]^

The study has some limitations. First, the reconstruction was conducted in a single medical center by only a craniomaxillofacial surgeon, whose personal experiences and preferences heavily influenced the reconstructive strategies. Second, nearly all of the patients in the cohort had medium-to large-sized defects, thus creating a bias in evaluating the outcome of treating the small-sized defect. Finally, this is a retrospective study with a small number of cases. Strict trials and more cases are required to provide higher levels evidence for guiding cranioplasty failure management.

## 5. Conclusions

The management of cranioplasty failure is challenging due to the high complication rate and the need for multiple reconstructive procedures. The reconstruction should follow the principles of the reconstructive ladder and the concept of “like with like” in order to achieve satisfying functional and aesthetic outcomes. Before establishing the reconstructive plan, the location and size of the defect, etiology, wound quality, infection, underlying tissue exposure, hairline involvement, and presence of skull deformity should all be carefully considered. A multidisciplinary team, including reconstructive and plastic surgeons, neurosurgeons, radiologists, psychiatrists, and a rehabilitation team, is essential to provide a comprehensive reconstructive program. Therefore, this study investigated the complications and the reconstructive procedures in patients with cranioplasty failure, with the goal of developing an algorithmic approach for optimal reconstructive strategies.

## Author contributions

**Conceptualization:** Yu-Chi Wang, Su-Shin Lee.

**Data curation:** Yu-Chi Wang.

**Formal analysis:** Yu-Chi Wang.

**Software:** Yu-Chi Wang.

**Visualization:** Yu-Chi Wang.

**Writing – original draft:** Yu-Chi Wang.

**Writing – review & editing:** Yi-Chia Wu, Chao-Wei Chang, Chia-Li Chung, Su-Shin Lee.
